# Comparative analysis of AI on human nutrition knowledge: Evaluating large language model-based conversational agents against dietetics students and the general population

**DOI:** 10.1371/journal.pone.0336577

**Published:** 2025-12-08

**Authors:** Nicola Luigi Bragazzi, Stefania Monica, Federico Bergenti, Francesca Scazzina, Alice Rosi

**Affiliations:** 1 Human Nutrition Unit, Department of Food and Drug, University of Parma, Parma, Italy; 2 Department of Sciences and Method for Engineering, University of Modena and Reggio Emilia, Reggio Emilia, Italy; 3 Department of Engineering for Industrial Systems and Technologies, University of Parma, Parma, Italy; Northern Border University, SAUDI ARABIA

## Abstract

Understanding the core principles of nutrition is essential in the contemporary context of abundant and often contradictory dietary advice, to empower individuals to make informed dietary choices and manage diet-related non-communicable diseases. The role of Artificial Intelligence (AI) in providing nutritional information is increasingly prominent, but its reliability in this domain is not well-established yet. This study compares the nutrition knowledge of state-of-the-art Large Language Model (LLM)-based conversational agents and chatbots with that of human subjects having different levels of nutrition knowledge. The “General Nutrition Knowledge Questionnaire–Revised” (GNKQ-R) was administered to four LLMs (ChatGPT-3.5, ChatGPT-4, Google Bard, currently known as Google Gemini, and Microsoft Copilot), using zero-shot prompts. Responses were scored in accordance with the GNKQ-R’s guidelines. The average performance of AI systems across all LLMs was 77.3 ± 5.1 out of 88, comparable to that of dietetics students and significantly higher than English students. ChatGPT-4 scored highest among the LLMs (82/88), surpassing both groups of students (dietetics: 79.3/88, English: 67.7/88) as well as all other demographic groups. In “Dietary Recommendations”, ChatGPT-3.5 and ChatGPT-4 demonstrated comparable performance to dietetics students. ChatGPT-4 excelled in “Food Groups”, outperforming all human groups. In “Healthy Food Choices”, ChatGPT-4 achieved a perfect score, indicating a deep understanding of this subject. ChatGPT-3.5 excelled in “Diet, Disease and Weight Management”. Variations in the performances of the LLMs across different sections were observed, suggesting knowledge gaps in certain areas. Some of the tested LLMs, particularly ChatGPT-3.5 and ChatGPT-4, showed proficiency in nutrition knowledge, rivaling or even surpassing dietetics students in certain sections. This indicates their potential utility in nutritional guidance. However, this study also identified nuances and specific details where LLMs lack compared to specialized human education. The study highlights the potential of AI in public health and educational settings. However, LLMs may be limited in their capacity to generate personalized dietary advice that accounts for clinical complexity and individual variability, reinforcing the indispensable role of expert human judgment.

## Introduction

Dietary patterns have a significant impact on human health, and diet-related risk factors are among the main preventable risk factors linked to the incidence of non-communicable diseases (NCDs) [1], causing 11 million deaths in 2017 [2]. Increasing the consumption of healthy diets can, therefore, improve public health and prevent chronic diseases. Proper nutrition knowledge is a crucial factor in promoting healthier food choices. Individuals who possess adequate nutrition knowledge are better equipped to make informed food choices, leading to improved health outcomes [3–5].

Proper general nutrition knowledge is not merely beneficial; it is a pivotal element in molding dietary behaviors and lifestyle choices, with far-reaching implications for individual well-being and overall public health [6]. In an era characterized by the proliferation of nutritional misinformation and disinformation, where dietary advice is abundant yet often contradictory [7,8], understanding the core principles of nutrition has become paramount. This foundational knowledge empowers individuals to navigate the complex landscape of dietary choices, facilitating informed decisions about their nutrition and health. Adequate knowledge of nutrition is pivotal in the prevention and management of prevalent NCDs, including obesity, diabetes, heart diseases, and certain cancers, which are significantly influenced by dietary habits [9]. Furthermore, it enables individuals to evaluate a multitude of diet trends and make choices that truly benefit their physical and mental health [10].

With an increasing number of individuals seeking dietary guidance through digital platforms [11], generative Artificial Intelligence (AI) has rapidly gained interest. Conversational agents and chatbots are specifically emerging as key sources of personalized nutritional information [12–15], given their ease of use and availability. In the rapidly evolving domain of AI, examining AI-based systems’ understanding in specialized fields such as nutrition is becoming particularly critical [16,17]. Yet, their capabilities to reliably address nutrition-related inquiries in the digital landscape where nutritional misinformation and disinformation are increasingly rampant remain largely unexplored. To date, only a limited number of studies [18–20] have been conducted to evaluate the proficiency of these platforms in responding to queries about nutrition. However, these investigations generally make use of non-validated questionnaires and do not compare the different existing AI systems.

It is crucial to thoroughly assess and validate the effectiveness and accuracy of AI-based responses, especially from a comparative perspective that looks at contrasting various AI systems. A variety of AI-based models exists from those developed by OpenAI (ChatGPT-3.5 and ChatGPT-4) to those devised by Microsoft (Copilot) and Google (Bard, currently known as Google Gemini). These Large Language Models (LLMs) are characterized by shared foundational elements, built on the Transformer architecture [21] and trained on diverse datasets encompassing texts from various sources, including publicly available books, websites, and other open-source resources. The training data for these models typically includes a wide array of material designed to provide a broad understanding of language and contextual knowledge. For instance, ChatGPT-3.5 and ChatGPT-4 were trained on datasets containing information up to September 2021, covering a broad spectrum of publicly available text to ensure contextual and domain versatility. Microsoft Copilot and Google Bard similarly rely on datasets gathered over comparable timeframes. This allows them to proficiently comprehend and generate human-like text, excelling in natural language processing (NLP) and mimicking engaging conversations. However, these LLMs vary in model size and outcomes, including performance, accuracy, and coherence. ChatGPT-3.5 is proficient in generating coherent responses across general topics but may struggle with highly specialized queries, while ChatGPT-4 builds on this by offering enhanced contextual understanding and accuracy, particularly in complex reasoning tasks. Microsoft Copilot streamlines document creation, summarization, and various workflow tasks through automation. In contrast, Google Bard complements Google Search by delivering up-to-date answers drawn from the internet, thereby allowing users to delve deeply into topics of interest, though it may still encounter limitations in handling specialized information. Each model’s unique design aligns with its intended use. Consequently, these LLMs may differ in their ability to process complex or ambiguous queries and generate contextually relevant responses within a specific context. Therefore, it is essential to compare these LLMs rather than focusing solely on one because understanding their strengths and weaknesses helps users choose the most appropriate tool for specific needs and promotes the advancement of AI technology. Analyzing each LLM’s areas of expertise and limitations enables researchers to identify opportunities for optimization, enhancement, and innovation in future iterations.

Currently, to the best of our knowledge, no comparative study appraising various LLMs in the field of human nutrition has been conducted. Therefore, the present study was undertaken with the aim of addressing this knowledge gap, by presenting an in-depth comparison of nutrition knowledge between state-of-the-art LLMs (ChatGPT-3.5, ChatGPT-4, Google Bard, and Microsoft Copilot) and human subjects, including students with diverse academic backgrounds, and various demographic groups (in terms of age, gender, education level, and health status). Employing the revised version of the “General Nutrition Knowledge Questionnaire” (GNKQ-R) as a standard [22], overall assessments and scores across the four principal sections of the questionnaire (namely, “Dietary Recommendations”, “Food Groups”, “Healthy Food Choices”, and “Diet, Disease and Weight Management”) were analyzed and contrasted.

The objective of this study was to evaluate the capability of the tested LLMs in comprehending and effectively communicating nutritional information, in comparison to the nuanced and comprehensive understanding possessed by human individuals. We hypothesized that these LLMs would achieve high scores on the GNKQ-R, surpassing English students and demonstrating a level of nutrition knowledge comparable to or exceeding that of dietetics students. More specifically, these LLMs are expected to excel in sections related to basic dietary recommendations, food group classifications, and general healthy food choices. However, they are anticipated to show variability in their performance across different sections, with potential deficiencies in areas requiring deep, specialized knowledge and practical application of nutrition principles, such as diet-disease relationships and weight management strategies. Last, despite their high overall scores, these LLMs are expected to exhibit occasional deficiencies in the nuanced understanding and context-specific expertise that human dietetics students possess.

## Methods

### Procedure

The GNKQ-R [22], the revised and updated version of the “General Nutrition Knowledge Questionnaire”, initially developed by Parmenter and Wardle in the nineties in the United Kingdom [23], and, subsequently, tested and validated in diverse populations [3], was employed to benchmark the LLMs.

The selection of the GNKQ-R as the primary tool for comparison in this study is grounded in its strong validation, reliability, and suitability for the research objectives. As a rigorously developed and widely recognized instrument, the GNKQ-R provides a standardized framework for assessing general nutrition knowledge, thereby ensuring the credibility and robustness of the study’s findings. The questionnaire’s comprehensive design allows it to evaluate multiple dimensions of nutrition knowledge. This breadth of coverage aligns with the study’s aim of conducting a detailed comparative analysis of human and LLM performance across these key areas. Additionally, the questionnaire’s relevance across a wide range of demographic and expertise levels allows for nuanced comparisons between LLMs and human groups, supporting the study’s goal of examining diverse knowledge levels.

Questions from the GNKQ-R [22] were submitted to ChatGPT-3.5, ChatGPT-4, Google Bard, and Microsoft Copilot, using zero-shot prompts. This means that the LLMs were queried without any prior specific training or examples given for the task at hand. In other words, these tools were asked to perform a task they have not explicitly been trained to do, using only their pre-existing knowledge and capabilities.

These four LLMs were chosen based on their availability at the time of our research (January 2024) and our research aims. Our purpose was to analyze conversational agents and chatbots like ChatGPT, which offer a user-friendly web interface accessible to individuals without coding expertise. This accessibility enables a broad spectrum of users, from beginners to advanced coders, to interact with these tools or integrate them into applications with minimal setup. In contrast, open-source and open-weight models like Mistral and LLaMA, while powerful, are geared more toward technically proficient users. They require coding knowledge for installation, fine-tuning, and deployment in programming environments like Python. Users of these models must set up infrastructure, manage dependencies, and often adapt the models for specific use cases. This makes Mistral and LLaMA highly flexible for developers, yet less accessible for non-technical users compared to the more user-friendly interface of ChatGPT. Consequently, models like Mistral and LLaMA are not directly comparable to ChatGPT in terms of accessibility and intended user base.

**Fig 1:**
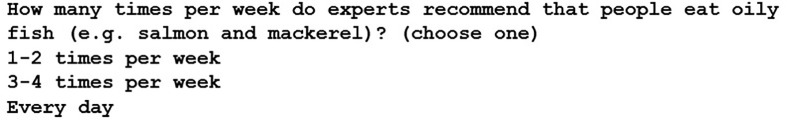
Example of the prompts used to query the tested Large Language Models (LLMs). The shown prompt was used to feed the LLMs with the fifth question of Section 1 of the “General Nutrition Knowledge Questionnaire–Revised” questionnaire.

All four selected LLMs were queried on the same day (January 10, 2024), using their available public web interface. To assess consistency, we repeated the testing process a week later using the same questions and prompts. The results showed identical scoring patterns, suggesting the models’ consistency in handling nutrition-related queries despite the potentially inherent variability in LLM performance. Fig 1 shows an example of the prompts used to administer the GNKQ-R to the tested LLMs. Note that the questions of the GNKQ-R were slightly rephrased to avoid references to visual elements (images and labels) and to the explicit request for ticking an answer. At the time of data collection, LLMs could not process visual elements as some multimodal systems can today. These adjustments were purely presentational and did not alter the substantive content or intended meaning of the questions, thereby preserving the validity of the instrument.

The full list of used prompts is reported in S1 File available from the website of the journal.

Replies were collected, transcribed into an Excel spreadsheet, and scored according to the instructions of the developers of the questionnaire. Points were assigned on a per-item basis, with each correct response earning one point, summing up to a potential maximum of 18 points for Section 1 (“Dietary Recommendations”), 36 points for Section 2 (“Food Groups”), 13 points for Section 3 (“Healthy Food Choices”), and 21 points for Section 4 (“Diet, Disease and Weight Management”), and up to 88 points for the total questionnaire. The full list of obtained answers is reported in S2 File available from the website of the journal.

### Human participants

Scores from the GNKQ-R questionnaire were collected from UK university students as reported in a previously published work [22]. This represents the most updated revised version of the tool, published in 2016, and no more recent updates currently exist. In this study, Kliemann et al. reported that 96 students from nutrition or dietetics courses (named “dietetics students”) and 89 students from English courses (named “English students”) from all over the UK completed the GNKQ-R questionnaire online on a single occasion. The two student groups were similar in terms of socio-demographic (age, gender, and socio-economic status) and clinical (health status) related parameters but were expected to be different in their nutrition knowledge.

In addition, data from students were analyzed together with the scores from 266 UK respondents (n = 185 from the general population and n = 81 from a charity supporting overweight individuals) for a total sample of 451 participants who completed the GNKQ-R [22]. Participants were categorized into three age groups: i) 18–35 years of age (n = 195), ii) 36–50 years (n = 108), and iii) 50 years and older (n = 148). Concerning gender, female participants were the majority (n = 335). Participants were also divided based on their highest level of education: i) no degree (including individuals with secondary school education, O levels to A levels, or a certificate/diploma, generally with lower nutrition knowledge scores, n = 239), and ii) degree or higher (including participants with a degree or postgraduate degree, n = 212). Finally, health status was self-reported and categorized into three groups: i) poor/fair health (n = 148), ii) good health (n = 183), and iii) very good/excellent health (n = 120).

### Ethical considerations

#### Human subject research ethics review.

This study analyzed published aggregate data [22]; no additional human data were collected. The study [22] from which data related to human participants were extracted received ethical approval from the Ethics Committee of University College London and the University of Surrey (UK). These data were not accessed individually in the present analysis; instead, we used published summary statistics data.

In contrast, ethical approval was not required for the LLM-related aspects of the present research since LLMs did not involve any human participants or the handling of personal, sensitive, or identifiable data. The study ensured that all LLM-related activities adhered to principles of transparency and responsibility, focusing solely on evaluating the LLM’s capabilities without breaching ethical or legal boundaries.

#### Informed consent.

Informed consent was obtained from all participants involved in the study [22] from which data related to human participants were extracted. For online data collection, consent was given when participants clicked “Next page” to proceed with the survey.

In contrast, informed consent was not required for the LLM-related aspects of the present research since no human participants, personal information, or sensitive data were involved in the interaction with the LLMs.

#### Privacy and confidentiality.

The study [22] from which data related to human participants were extracted adhered to strict privacy and confidentiality measures. All collected data were anonymized or de-identified before analysis. Personal identifiers were removed to prevent the possibility of re-identification, ensuring compliance with data protection standards.

In contrast, privacy and confidentiality concerns were not applicable to the LLM-related aspects of this research since the tested LLMs were queried using publicly accessible interfaces without involving any personal or sensitive data. The interactions with the LLMs were limited to standardized questions from the GNKQ-R, ensuring that no identifiable or private information was used, processed, or stored during the study.

#### Participant compensation.

Participants in the study [22] were provided with different forms of compensation as part of their involvement. Participants from Research Now’s panel received incentives in the form of e-Rewards points, which could be redeemed for various rewards. Participants recruited from Weight Concern’s panel did not receive compensation. University students were entered into a prize draw for a £25 high-street voucher.

In contrast, compensation was not applicable to the LLM-related aspects of this research since the interaction with LLMs involved querying the tools using publicly available interfaces, without engaging human participants or requiring time, effort, or data contribution from individuals.

### Statistical analysis

Descriptive statistics of the overall assessments and the scores broken down according to each section for each LLM were carried out. LLM proficiency was benchmarked against different populations. Students from both nutritional (dietetics students) and non-nutritional (English students) backgrounds were chosen as examples of a population with a higher general knowledge of nutrition (dietetics students), i.e., a best-case scenario, and as a population of the same age and socio-cultural status that is comparable to the general population in terms of nutritional knowledge (English students), respectively. These populations were pooled together with members of the general population, to compare various demographic groups (in terms of age, gender, education, and health status). Then, the performances of the four LLMs were averaged to provide a comprehensive overview of their collective capabilities, rather than focusing solely on the peak performance of a single LLM. This approach ensured that the evaluation reflected the general proficiency of AI in nutrition knowledge, capturing variability across different LLMs and avoiding bias from exceptional results. It also allowed for a fair and robust comparison with the performances of dietetics and English students and the various demographics. These data were extracted from [22] and compared by carrying out an Analysis of Variance (ANOVA) from summary statistics and using the Tukey Honest Significant Difference (HSD) test for the post-hoc analysis.

All statistical analyses were done using the open-source R environment (R Core Team, R: A Language and Environment for Statistical Computing, R Foundation for Statistical Computing, Vienna, Austria, 2024).

All the underlying data and the code have been deposited into Zenodo and can be publicly accessed at https://doi.org/10.5281/zenodo.17166244.

## Results

### Large language models versus humans

We present the findings from the comparative performance analysis conducted in four stages. First, as previously mentioned, we benchmarked the proficiency of each LLM against dietetics and English students. In the second stage, we broadened the comparison to include a diverse cohort of individuals, comprising both students and members of the general population, stratified by various demographic characteristics. This approach mirrors the methodology of the study [22], from which data on human participants were extracted, and was designed to facilitate easier, more effective, and direct comparisons of the findings. After evaluating the performance of each LLM individually, in the final two stages, an average score was calculated to provide a comprehensive measure of their capabilities against dietetics and English students (third stage) and against the pooled cohort of human participants (fourth stage).

### Large language models versus dietetics and english students

**Fig 2:**
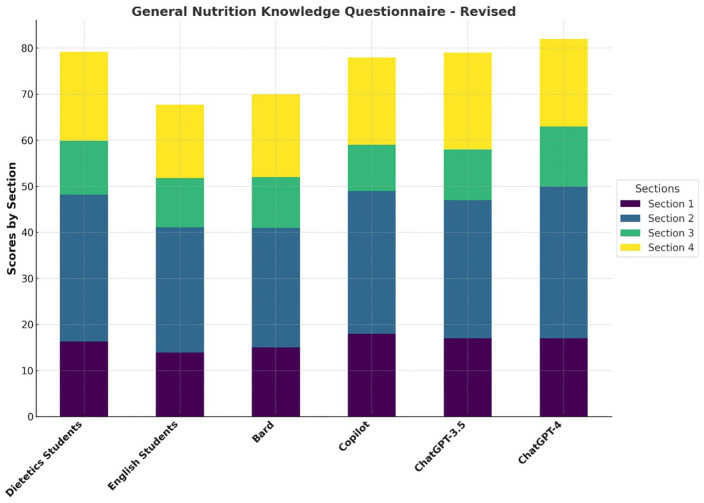
Comparative performance analysis on the “General Nutrition Knowledge Questionnaire–Revised” (GNKQ-R). This chart illustrates the scores of dietetics and English students versus various Large Language Models (Google Bard, Microsoft Copilot, ChatGPT-3.5, and ChatGPT-4) across the four key sections of the GNKQ-R: “Dietary Recommendations” (Section 1), “Food Groups” (Section 2), “Healthy Food Choices” (Section 3), and “Diet, Disease and Weight Management” (Section 4).

Findings from the comparative performance analysis on the GNKQ-R questionnaire are presented in Fig 2 for the total score and by sections across the different study groups.

In the validation study dietetics and English students had an average score of 79.3/88 and 67.7/88, respectively [22]. Among the LLMs, ChatGPT-4 led with 82/88, indicating a robust understanding of nutrition topics. ChatGPT-3.5 demonstrated a competitive understanding with a score of 79/88. Google Bard and Microsoft Copilot showed intermediate performances with scores of 70/88 and 78/88.

Concerning “Dietary recommendations” (Section 1), ChatGPT-4 and ChatGPT-3.5 both scored 17/18, nearly mirroring the dietetics students (16.3/18), thus reflecting strong foundational knowledge. Microsoft Copilot excelled with 18/18, suggesting a superior understanding in this section. Google Bard and English students scored lower, 15/18 and 13.9/18 respectively, indicating knowledge gaps.

In terms of the knowledge of “Food groups” (Section 2), ChatGPT-4 outperformed all groups with 33/36, including dietetics students (31.9/36), demonstrating a high level of proficiency. Microsoft Copilot and ChatGPT-3.5 had close scores of 31/36 and 30/36. Google Bard and English students lagged behind, scoring 26/36 and 27.2/36, respectively.

Regarding Section 3, ChatGPT-4 achieved a perfect score of 13/13, indicating a deep understanding of “Healthy food choices”, while dietetics students scored 11.7/13, showing strong, but not flawless, knowledge. ChatGPT-3.5, Google Bard, and English students had similar scores (11/13, 11/13, and 10.7/13), suggesting moderate understanding. Microsoft Copilot scored the lowest in this category with 10/13.

Finally, concerning “Diet, disease and weight management” (Section 4), ChatGPT-3.5 excelled with a perfect score of 21/21. ChatGPT-4 and Microsoft Copilot both scored 19/21, showing strong competency, similar to the one of dietetics students (19.3/21). Bard scored 18/21, indicating good, but not perfect, knowledge. English students scored the worst (15.9/21).

In general, as shown in Fig 2, ChatGPT-4 outperformed both groups of students in the overall score and in most individual sections. ChatGPT-3.5 had scores similar to ChatGPT-4 in some sections and also demonstrated a strong overall performance, similar to the one of the dietetics students. Google Bard and Microsoft Copilot showed competitive but varying performance across different sections, and Google Bard displayed an overall performance like not trained students.

### Large language models versus dietetics and english students and the general population

**Fig 3:**
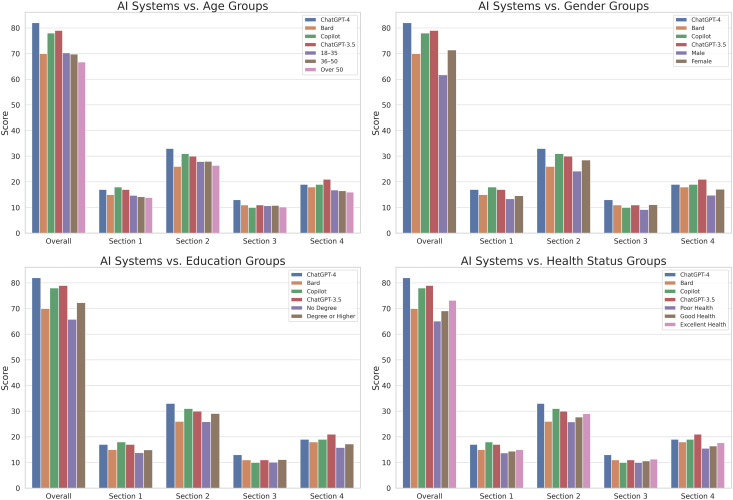
Comparative analysis of the “General Nutrition Knowledge Questionnaire–Revised” (GNKQ-R) scores. This set of four bar charts illustrates the performance of the Large Language Models (ChatGPT-3.5, ChatGPT-4, Google Bard, and Microsoft Copilot) against different demographic groups categorized by age, gender, education level, and health status. Each chart provides a detailed breakdown of scores across ‘Overall’ performance and individual sections (1 to 4) of the GNKQ-R, highlighting the proficiency levels in each category.

The comparative performance analysis on the GNKQ-R questionnaire total score and section sub-scores is presented in Fig 3 for age groups, gender groups, education level groups, and health status groups. These results were derived from the pooled cohort of students and members of the general population, providing a comprehensive overview of performance across diverse demographic categories. ChatGPT-4 outperformed all demographic groups in the overall score (82/88 versus 61.7/88 in males and 71.4/88 in females; 82/88 versus 65.8/88 in the no degree group and 72.3/88 in the group with degree or higher; 82/88 versus 70.3/88 in the group aged 18–35 years, 69.8/88 in the group aged 36–50 years, and 66.7/88 in the group aged 50 years and greater; 82/88 versus 65.1/88 in the poor health group, 69.1/88 in the good health group, and 73.2/88 in the very good/excellent health group).

ChatGPT-4 outperformed as well in all individual sections of the GNKQ-R questionnaire.

ChatGPT-3.5 had scores similar to ChatGPT-4 in some sections and also demonstrated a strong overall performance in comparison to all demographic groups, being slightly outperformed only in Section 3 by females (11/13 versus 11.1/13) and the very good/excellent health group (11.3/13).

Finally, Google Bard and Microsoft Copilot showed competitive but varying performance across different sections. In terms of the overall score, Google Bard was outperformed by female participants (71.4/88 versus 70/88), the group with degree or higher (72.3/88), the very good/excellent health status group (73.2/88), and the group aged 18–35 years (70.3/88). While exhibiting strong scores in Section 1 and Section 4, Google Bard was weaker in Section 2 and Section 3. Microsoft Copilot demonstrated a very good overall score, and outperformed all demographics in sections 1, 2, and 4, demonstrating a weaker performance for Section 3.

### LLM overall performance versus dietetics and english students

For statistical purposes, the performances of the four LLMs were averaged to be compared against those of dietetics and English students. LLM overall mean score was 77.3 ± 5.1. For the “Dietary Recommendations” section the mean score was 16.8 ± 1.3, in the “Food Groups” category the mean was 30.0 ± 2.9 and the “Healthy Food Choices” section had a mean score of 11.3 ± 1.3. Lastly, the “Diet, Disease and Weight Management” category showed a mean of 19.3 ± 1.3. At the ANOVA, mean scores of the performances of all LLMs and dietetics students did not differ in a statistically significant way (both overall and for each section), while they differed from the scores achieved by English students (overall, with a *P*-value of.03, and for the sections 1 and 4, with *P*-values of.01 and.005, respectively) (Table 1).

**Table 1:** Findings from ANOVA and post-hoc analyses comparing the average performance of the Large Language Models against dietetics and English students.

GNKQ-R	Overall ANOVA	Post-hoc analyses	
		Averaged AI *vs* dietetics students	Averaged AI *vs* English students
Overall GNKQ-R	F = 58.8, *P* < .001	Diff = −2.0, 95%CI = −10.8 to 6.8, *P* = .85	Diff = 9.6, 95%CI = 0.8 to 18.4, *P* = .03
Section 1	F = 36.9, *P* < .001	Diff = 0.5, 95%CI = −1.8 to 2.8, *P* = .87	Diff = 2.9, 95%CI = 0.6 to 5.2, *P* = .01
Section 2	F = 37.5, *P* < .001	Diff = −1.9, 95%CI = −6.4 to 2.6, *P* = .57	Diff = 2.8, 95%CI = −1.7 to 7.3, *P* = .30
Section 3	F = 8.4, *P* < .001	Diff = −0.4, 95%CI = −2.4 to 1.6, *P* = .88	Diff = 0.6, 95%CI = −1.4 to 2.6, *P* = .76
Section 4	F = 62.2, *P* < .001	Diff = 0.0, 95%CI = −2.5 to 2.5, *P* = 1.00	Diff = 3.4, 95%CI = 0.9 to 5.9, *P* = .005

### LLM overall performance versus dietetics and english students and the general population

Concerning the other demographics, the average performance of the LLMs passed the performance of male participants in terms of the overall score (*P* = .02), Section 1 (*P* = .001), Section 2 (*P* = .02), Section 4 (*P* < .001), and Section 3 in a borderline way (*P* = .08). Performance was comparable with female participants, showing a trend toward higher scores in Section 1 (*P* = .06) and a more modest advantage in Section 4 (*P* = .13), however without achieving statistical significance in both cases (Table 2).

**Table 2:** Findings from ANOVA and post-hoc analyses comparing the average performance of the Large Language Models against male and female participants.

GNKQ-R	Overall ANOVA	Post-hoc analyses	
		Averaged AI *vs* males	Averaged AI *vs* females
Overall GNKQ-R	F = 34.0, *P* < .001	Diff = 15.6, 95%CI = 2.3 to 28.9, *P* = .02	Diff = 5.9, 95%CI = −7.2 to 19.0, *P* = .54
Section 1	F = 20.5, *P* < .001	Diff = 3.4, 95%CI = 1.1 to 5.7, *P* = .001	Diff = 2.2, 95%CI = −0.1 to 4.5, *P* = .06
Section 2	F = 46.1, *P* < .001	Diff = 5.8, 95%CI = 0.8 to 10.8, *P* = .02	Diff = 1.5, 95%CI = −3.5 to 6.5, *P* = .76
Section 3	F = 43.0, *P* < .001	Diff = 2.1, 95%CI = −0.2 to 4.4, *P* = .08	Diff = 0.2, 95%CI = −2.1 to 2.5, *P* = .98
Section 4	F = 48.6, *P* < .001	Diff = 4.5, 95%CI = 1.8 to 7.2, *P* < .001	Diff = 2.2, 95%CI = −0.5 to 4.9, *P* = .13

Compared with participants with degrees or higher titles (Table 3), the average performance of the LLMs was similar, with the exception of Section 1 (*P* = .03) where it was higher. It outperformed participants with no degree in Section 1 (*P* < .001) and demonstrated a borderline trend toward nominal significance in Section 4 (*P* = .05). An additional borderline post-hoc trend was observed for the overall GNKQ-R score when compared with participants without a degree (P = .11).

**Table 3:** Findings from ANOVA and post-hoc analyses comparing the average performance of the Large Language Models against participants of various education groups.

GNKQ-R	Overall ANOVA	Post-hoc analyses	
		Averaged AI *vs* no degree	Averaged AI *vs* degree or higher
Overall GNKQ-R	F = 19.4, *P* < .001	Diff = 11.5, 95%CI = −2.0 to 25.0, *P* = .11	Diff = 5.0, 95%CI = −8.5 to 18.5, *P* = .66
Section 1	F = 35.7, *P* < .001	Diff = 3.0, 95%CI = 1.2 to 4.8, *P* < .001	Diff = 1.9, 95%CI = 0.1 to 3.7, *P* = .03
Section 2	F = 20.1, *P* < .001	Diff = 4.1, 95%CI = −2.3 to 10.5, *P* = .29	Diff = 0.9, 95%CI = −5.5 to 7.3, *P* = .94
Section 3	F = 25.5, *P* < .001	Diff = 1.2, 95%CI = −0.6 to 3.0, *P* = .25	Diff = 0.2, 95%CI = −1.6 to 2.0, P = .96
Section 4	F = 14.0, *P* < .001	Diff = 3.5, 95%CI = −0.1 to 7.1, *P* = .05	Diff = 2.1, 95%CI = −1.5 to 5.7, *P* = .35

Finally, no differences could be found when comparing the LLMs against human participants in terms of age (Table 4), although some post-hoc comparisons showed borderline trends: in Section 1 for participants aged 36–50 years (P = .18) and >50 years (P = .11).

**Table 4:** Findings from ANOVA and post-hoc analyses comparing the average performance of the Large Language Models against participants of various age groups.

GNKQ-R	Overall ANOVA	Post-hoc analyses		
		Averaged AI *vs* 18–35 years	Averaged AI *vs* 36–50 years	Averaged AI *vs* > 50 years
Overall GNKQ-R	F = 3.3, *P* = 0.02	Diff = 7.0, 95%CI = −8.7 to 22.7, *P* = .66	Diff = 7.5, 95%CI = −8.3 to 23.3, *P* = .61	Diff = 10.6, 95%CI = −5.2 to 26.4, *P* = .31
Section 1	F = 4.2, *P* = .006	Diff = 2.1, 95%CI = −1.2 to 5.4, *P* = .35	Diff = 2.6, 95%CI = −0.7 to 5.9, *P* = .18	Diff = 2.9, 95%CI = −0.4 to 6.2, *P* = .11
Section 2	F = 2.6, *P* = .049	Diff = 2.1, 95%CI = −5.4 to 9.6, *P* = .89	Diff = 2.0, 95%CI = −5.5 to 9.5, *P* = .90	Diff = 3.6, 95%CI = −3.9 to 11.1, *P* = .60
Section 3	F = 2.2, *P* = .09	Diff = 0.6, 95%CI = −2.3 to 3.5, *P* = .95	Diff = 0.5, 95%CI = −2.4 to 3.4, *P* = .97	Diff = 1.1, 95%CI = −1.8 to 4.0, *P* = .75
Section 4	F = 2.5, *P* = .06	Diff = 2.5, 95%CI = −1.9 to 6.9, *P* = .46	Diff = 2.8, 95%CI = −1.6 to 7.2, *P* = .36	Diff = 3.3, 95%CI = −1.1 to 7.7, *P* = .22

Similarly, no statistically significant differences emerged when comparing GNKQ-R performance across health status groups (Table 5). The only results approaching borderline significance concerned comparisons between averaged AI and poor health status in the overall GNKQ-R (P = .19), Section 1 (P = .07), and Section 4 (P = .10).

**Table 5:** Findings from ANOVA and post-hoc analyses comparing the average performance of the Large Language Models against participants of various health status groups.

GNKQ-R	Overall ANOVA	Post-hoc analyses		
		Averaged AI *vs* poor health status	Averaged AI *vs* good health status	Averaged AI *vs* excellent health status
Overall GNKQ-R	F = 10.7, *P* < .001	Diff = 12.2, 95%CI = −3.6 to 28.0, *P* = .19	Diff = 8.2, 95%CI = −7.5 to 23.9, *P* = .54	Diff = 4.1, 95%CI = −11.7 to 19.9, *P* = .91
Section 1	F = 7.4, *P* < .001	Diff = 3.1, 95%CI = −0.2 to 6.4, *P* = .07	Diff = 2.4, 95%CI = −0.8 to 5.6, *P* = .22	Diff = 1.8, 95%CI = −1.5 to 5.1, *P* = .48
Section 2	F = 7.6, *P* < .001	Diff = 4.2, 95%CI = −3.2 to 11.6, *P* = .46	Diff = 2.3, 95%CI = −5.1 to 9.7, *P* = .85	Diff = 1.0, 95%CI = −6.4 to 8.4, *P* = .99
Section 3	F = 8.3, *P* < .001	Diff = 1.3, 95%CI = −1.5 to 4.1, *P* = .63	Diff = 0.7, 95%CI = −2.1 to 3.5, *P* = .92	Diff = 0.0, 95%CI = −2.8 to 2.8, *P* = 1.00
Section 4	F = 11.2, *P* < .001	Diff = 3.8, 95%CI = −0.4 to 8.0, *P* = .10	Diff = 2.9, 95%CI = −1.3 to 7.1, *P* = .29	Diff = 1.6, 95%CI = −2.7 to 5.9, *P* = .77

## Discussion

In the contemporary era, characterized by the widespread dissemination of misinformation and disinformation regarding nutrition, the significance of proper nutrition knowledge in shaping dietary behaviors and health outcomes cannot be overstated. With the rise of digital platforms as sources of dietary guidance, AI-based tools like conversational agents and LLMs are becoming increasingly popular for providing personalized nutritional information [24,25]. However, the reliability of AI in addressing nutrition-related inquiries is not well-established, as evidenced by the limited number of studies that have evaluated its proficiency in nutrition, often without the utilization of validated methodologies or comprehensive comparisons across different LLMs.

To address this gap in the literature, this comparative analysis of the scores in each section of a validated questionnaire assessing the general nutrition knowledge was undertaken to offer valuable insights into the present capabilities and potential roles of AI in the fields of nutrition and dietetics.

The performance of the tested LLMs, specifically ChatGPT-4, demonstrated a high level of proficiency, often matching or exceeding that of dietetics students in certain evaluated sections. This finding suggests that these LLMs have robust, up-to-date nutrition knowledge, which can be valuable in disseminating nutrition information, with ChatGPT-4 showing high competence in providing nutrition-related information and being better aligned with established nutritional guidelines.

This finding aligns with those of a small number of existing studies that have demonstrated that AI-based tools, such as ChatGPT, hold promise as valuable tools for addressing frequently posed nutrition queries to dietitians, offering supportive evidence for the potential utility of conversational agents in delivering nutritional guidance [18–20]. Haman and coworkers [18] showed that ChatGPT was highly accurate and consistent in providing nutritional estimations, particularly in energy values (97% within a 40% range of the data from the United States Department of Agriculture, USDA), and efficient in devising daily meal plans that aligned closely with USDA caloric values. Kirk and colleagues [19] solicited the most frequent nutrition-related queries from dietitians along with their expert responses. These queries were subsequently posed to ChatGPT, and both sets of replies were then evaluated by a panel of eighteen dietitians and nine subject matter experts for scientific accuracy, practical applicability, and clarity. ChatGPT’s responses attained higher overall scores compared to those from dietitians in five out of eight questions. Specifically, ChatGPT outperformed on scientific accuracy in five instances, on practical applicability in four, and on clarity in five. In comparison, the dietitians’ responses did not surpass ChatGPT’s average scores for any question, whether overall or in individual grading criteria. Last, Ponzo and colleagues [20] tested the ability of ChatGPT in providing nutritional guidelines in relation to different NCDs, comparing the responses given by ChatGPT to the most up-to-date nutritional guidelines. ChatGPT showed to be accurate in giving nutritional recommendations for specific diseases, with better performance for non-alcoholic fatty liver disease, type 2 diabetes, hypercholesterolemia, and hypertriglyceridemia. In addition, authors examined the performance of ChatGPT in providing personalized dietary advice in patients with multiple NCDs. In this case, the weaknesses of the LLM emerged, underlining its inadequacy in complex situations in which personalized nutritional strategies are urgently needed. However, these studies were limited to ChatGPT alone and did not explore other LLMs.

To the best of our knowledge, this study is the first to test and compare several LLMs using a validated nutrition knowledge questionnaire to assess their ability to provide proper nutrition information and advice for the general population. Our findings show variation in the performances of LLMs across various nutrition-related sections, which indicates that while the tested LLMs demonstrate a high level of knowledge, there are areas where they can still improve or where they lack the nuanced understanding that specialized human education provides. The high scores in “Healthy Food Choices” and “Diet, Disease and Weight Management” sections achieved by some LLMs suggest their potential utility in public health and educational settings, especially for providing general knowledge dissemination and potentially assist health professionals.

It is important to note that common errors have been observed in LLMs, with errors mostly concerning the classification of specific food group, and the relationship between diet and certain health conditions. These results suggest that while AI can be highly knowledgeable in nutrition, there are specific situations and details that may be challenging.

On the other hand, the differences between the two tested versions of ChatGPT (3.5 and 4) indicate advancements in AI capabilities over time, with newer versions showing improved accuracy in nutrition-related knowledge.

### Future directions

The rapid advancement and extensive adoption of LLMs like ChatGPT herald a new era in various fields, including nutrition and dietetics. As these technologies evolve, they are poised to become invaluable assistants for health professionals, offering quick information retrieval, draft text generation, and educational content creation. However, their limitations in accuracy, bias, and depth of understanding underscore the irreplaceable value of human expertise and judgment. Looking ahead, the integration of AI into professional practice must be approached with caution and a focus on complementing rather than replacing human knowledge, skills, and expertise. It is reasonable to hypothesize that in the future there will be a collaborative synergy between AI tools, including LLMs, and human health professionals, enhancing efficiency and accessibility while maintaining the quality and personalization of services, even for the most complex cases. Education and continuous training will play a crucial role in equipping professionals with the skills to effectively utilize these technologies, ensuring they remain at the forefront of their respective fields [15].

Future educational tools, particularly those leveraging Retrieval-Augmented Generation (RAG), may further enhance the effectiveness and reliability of AI-assisted learning by improving the accuracy and relevance of information retrieval. Integrating RAG techniques could play a pivotal role in expanding the scope and applicability of AI in nutrition education [26].

Moreover, future research should consider the collection of new, contemporary data from human participants to strengthen comparative analyses. This is particularly relevant as, in recent years, students and young adults have become increasingly aware of nutrition-related issues through digital health campaigns, social media, and curricular changes, potentially shifting baseline levels of nutrition knowledge compared to the cohorts examined in earlier validation studies. Expanding studies with updated samples would allow a more accurate benchmarking of AI systems against present-day standards of human knowledge.

### Strengths and limitations

The present study has some strengths, which should be properly acknowledged, including its novelty, its methodological rigor, and the systematic appraisal of major LLMs using a validated questionnaire. However, this study suffers from some limitations. Firstly, being a cross-sectional survey, it does not capture ongoing development and refinement in LLMs. Moreover, only general nutrition knowledge was tested: therefore, the findings cannot be generalized to situations in which highly specialized professional advice may be needed, especially in cases of medical nutrition therapy or specific dietary needs. It should also be noted that the GNKQ-R predominantly measures factual and procedural recall. While this is an important dimension of competence, it does not assess more advanced forms of reasoning that are critical in clinical and pharmaceutical nutrition practice. For example, the instrument cannot capture whether an LLM is able to synthesize dietary recommendations in the context of multimorbidity, anticipate interactions with complex pharmacological regimens, or weigh ethical and contextual considerations in patient-centered care. As such, the study provides insights into the knowledge retrieval capacity of LLMs, but does not evaluate their ability to generate safe, personalized, and clinically nuanced advice.

Furthermore, the validation of the GNKQ-R was published five years prior to the most recent training timeframe of the LLMs. Although no updated tools for assessing general nutrition knowledge may currently exist, and the GNKQ-R remains widely used to explore this construct [27,28], its use could introduce bias that may impact the benchmarking and evaluation of LLMs. This concern is further amplified by the temporal limitations of LLM training data. Unlike human professionals who continuously update their knowledge through ongoing education and practice, the tested LLMs are constrained by static training cut-offs (a limitation partially overcome by currently available LLMs). Nutrition science is a rapidly evolving discipline, with frequent revisions of clinical guidelines, new randomized controlled trials, and emerging debates around supplementation, dietary patterns, and chronic disease prevention. Consequently, an LLM trained only up to 2021 may not reflect current standards of care, thereby limiting both the validity and applicability of its outputs.

Another major limitation of the present study is the relatively small sample size of LLMs evaluated, which limits the generalizability of results to LLMs beyond those tested. Although the selected LLMs represent relatively distinct algorithms and knowledge bases reflective of available LLMs at the time, they may not encompass the full range of AI capabilities in nutrition, a constantly evolving field, as previously mentioned. Additionally, unequal sample sizes between LLMs and human demographic groups may introduce bias, reduce the accuracy of comparisons, and impact the ability to detect subtle performance differences.

Finally, an additional potential limitation of our study concerns the decision to average the performances of the four LLMs. While this approach provided a comprehensive indicator of how mainstream LLMs collectively perform relative to human groups (dietetics students, English students, and the general population), facilitated benchmarking, and avoided overemphasis on the strengths of a single model, it inevitably entails an assumption of comparability. The four models were tested under identical conditions and within the same time period to minimize confounding by access dates, prompt design, or system updates, yet averaging across heterogeneous systems may obscure important differences between them. For this reason, we reported both individual model results and the aggregate analysis: the individual results provide transparency regarding model-specific performance, while the averaged score allows for population-level statistical comparison with human cohorts.

Future research should increase the number of LLMs assessed and balance group sizes, stratifying analyses by model type or architecture to capture heterogeneity more comprehensively. Also, it should adopt more sophisticated outcome measures that extend beyond knowledge recall. These could include scenario-based assessments, longitudinal designs to capture model updates, and evaluation frameworks explicitly addressing safety, clinical integration, and ethical use in nutrition practice.

## Conclusions

The present comparison highlights the advancements in AI in understanding complex subjects like nutrition and its potential role in providing general information, or as an adjunct educational tool, in an era where digital and AI-based sources are increasingly sought for health-related guidance. At the same time, we assessed LLMs’ competence only in general nutrition knowledge, and our findings should not be extrapolated to contexts requiring individualized, clinically nuanced, or therapeutic advice. It remains essential to recognize the irreplaceable value of specialized human education and expertise: dietitians, pharmacists, and physicians are indispensable in interpreting AI-generated information and applying it within a full clinical context. Accordingly, AI should be regarded as a supportive, complementary resource for education and public engagement in nutrition, rather than as a substitute for professional judgment.

## Supporting information

S1 File Prompts used to query each Large Language Model. (DOCX)

S2 File Replies provided by Large Language Models. (DOCX)

## Abbreviations

AI: artificial intelligence
GNKQ-R: general nutrition knowledge questionnaire–revised
LLMs: large language models
NCDs: non-communicable diseases.
