# Supplementary material for: Comparative analysis of AI on human nutrition knowledge: Evaluating large language model-based conversational agents against dietetics students and the general population
Source: PLoS One. 2025 Dec 8;20(12):e0336577. doi: 10.1371/journal.pone.0336577 (PMC12685196; doi:10.1371/journal.pone.0336577)
Supplement: S1 File — (DOCX) [file pone.0336577.s001.docx]

**Supplementary Information 1. Prompts used to query each Large Language Model.**

Do health experts recommend that people should be eating more, the same amount, or less of the following foods? (choose one of the following three options “More”, “Same”, or “Less” per food)

- Fruit
- Food and drinks with added sugar
- Vegetables
- Fatty foods
- Processed red meat
- Wholegrains
- Salty foods
- Water

How many servings of fruit and vegetables per day do experts advise people to eat as a minimum? (One serving could be, for example, an apple or a handful of chopped carrots) (choose one)

- 2
- 3
- 4
- 5 or more

Which of these types of fats do experts recommend that people should eat less of? (choose one of the following two options “Eat less” or “Not eat less” per food)

- Unsaturated fats
- Trans fats
- Saturated fats

Which type of dairy foods do experts say people should drink? (choose one)

- Full fat (e.g. full fat milk)
- Reduced fat (e.g. skimmed and semiskimmed milk)
- Mixture of full fat and reduced fat
- Neither, dairy foods should be avoided

How many times per week do experts recommend that people eat oily fish (e.g. salmon and mackerel)? (choose one)

- 1-2 times per week
- 3-4 times per week
- Every day

Approximately how many alcoholic drinks is the maximum recommended per day (The exact number depends on the size and strength of the drink)? (choose one)

- 1 drink each for men and women
- 2 drinks each for men and women
- 2 drinks for men and 1 drink for women
- 3 drinks for men and 2 drinks for women

How many times per week do experts recommend that people eat breakfast? (choose one)

- 3 times per week
- 4 times per week
- Every day

If a person has two glasses of fruit juice in a day, how many of their daily fruit and vegetable servings would this count as? (choose one)

- One serving
- Two servings
- Three servings

According to the ‘eatwell guide’ (a guideline showing the proportions of food types people should eat to have a balanced and healthy diet), how much of a person’s diet should be made up of starchy foods? (choose one)

- Quarter
- Third
- Half

Do you think these foods and drinks are typically high or low in added sugar? (choose one of the following two options “High in added sugar” or “Low in added sugar” per food/drink)

- Diet cola drinks
- Natural yoghurt
- Ice cream
- Tomato ketchup
- Melon

Do you think these foods are typically high or low in salt? (choose one of the following two options “High in salt” or “Low in salt” per food)

- Breakfast cereals
- Frozen vegetables
- Bread
- Baked beans
- Red meat
- Canned soup

Do you think these foods are typically high or low in fibre? (choose one of the following two options “High in fibre” or “Low in fibre” per food)

- Oats
- Bananas
- White rice
- Eggs
- Potatoes with skin
- Pasta

Do you think these foods are a good source of protein? (choose one of the following two options “Good source of protein” or “Not a good source of protein” per food)

- Poultry
- Cheese
- Fruit
- Baked beans
- Butter
- Nuts

Which of the following foods do experts count as starchy foods? (choose one of the following two options “Starchy food” or “Not a starchy food” per food)

- Cheese
- Pasta
- Potatoes
- Nuts
- Plantains

Which is the main type of fat present in each of these foods? (choose one of the following four options “Polyunsaturated fat”, “Monounsaturated fat”, “Saturated fat”, or “Cholesterol” per food)

- Olive oil
- Butter
- Sunflower oil
- Eggs

Which of these foods has the most trans-fat? (choose one)

- Biscuits, cakes and pastries
- Fish
- Rapeseed oil
- Eggs

The amount of calcium in a glass of whole milk compared to a glass of skimmed milk is: (choose one)

- About the same
- Much higher
- Much lower

Which one of the following nutrients has the most calories for the same weight of food? (choose one)

- Sugar
- Starchy
- Fibre/roughage
- Fat

Compared to minimally processed foods, processed foods are: (choose one)

- Higher in calories
- Higher in fibre
- Lower in salt

If a person wanted to buy a yogurt at the supermarket, which would have the least sugar/sweetener? (choose one)

- 0% fat cherry yogurt
- Natural yogurt
- Creamy fruit yogurt

If a person wanted soup in a restaurant or cafe, which one would be the lowest fat option? (choose one)

- Mushroom risotto soup (field mushrooms, porcini mushrooms, arborio rice, butter, cream, parsley and cracked black pepper)
- Carrot butternut and spice soup (carrot, butternut squash, sweet potato, cumin, red chillies, coriander seeds and lemon)
- Cream of chicken soup (British chicken, onions, carrots, celery, potatoes, garlic, sage, wheat flour, double cream)

Which would be the healthiest and most balanced choice for a main meal in a restaurant? (choose one)

- Roast turkey, mashed potatoes and vegetables
- Beef, Yorkshire pudding and roast potatoes
- Fish and chips served with peas and tartar sauce

Which would be the healthiest and most balanced sandwich lunch? (choose one)

- Ham sandwich + fruit + blueberry muffin + fruit juice
- Tuna salad sandwich + fruit + low fat yogurt + water
- Egg salad sandwich + crisps + low fat yogurt + water

Which of these foods would be the healthiest choice for a pudding? (choose one)

- Berry sorbet
- Apple and blackberry pie
- Lemon cheesecake
- Carrot cake with cream cheese topping

Which of these combinations of vegetables in a salad would give the greatest variety of vitamins and antioxidants? (choose one)

- Lettuce, green peppers and cabbage
- Broccoli, carrot and tomatoes
- Red peppers, tomatoes and lettuce

If a person wanted to reduce the amount of fat in their diet, but didn’t want to give up chips, which of the following foods would be the best choice? (choose one)

- Thick cut chips
- Thin cut chips
- Crinkle cut chips

One healthy way to add flavour to food without adding extra fat or salt is to add: (choose one)

- Coconut milk
- Herbs
- Soya sauce

Which of the following cooking methods requires fat to be added? (choose one)

- Grilling
- Steaming
- Baking
- Sautéing

Traffic lights are often used on nutrition labelling, what would amber mean for the fat content of a food? (choose one)

- Low fat
- Medium fat
- High in fat

“Light” foods (or Diet foods) are always good options because they are low in calories. (choose one)

- Agree
- Disagree

Consider the following two products. Product 1 is a sweet biscuit. Each biscuit (9.5g) contains: calories 43 (2%), sugar 2g (2%), fat 1g (2%), saturates 1g (3%), salt 0.1g (2%). Typical value (as sold) per 100g is 450 kcal. Ingredient list includes oat flakes, sugar, palm oil, fortified wheat flour, whole wheat flour, fructose, malt syrup, salt, raising agents, sodium hydrogen carbonate, ammonium hydrogen carbonate, flavouring. Product 2 is a savoury biscuit. Each biscuit (16g) contains: calories 66 (3%), sugar 1g (1%), fat 3g (4%), saturates trace (1%), salt 0.3g (4%). Typical value (as sold) per 100g is 412 kcal. Ingredient list includes wheat flour, palm oil, corn syrup, malt, salt, yeast, leavening agents (sodium bicarbonate, ammonium bicarbonate, sodium pyrophosphate), corn starch, soy lecithin, sodium metabisulphite (baking agent). Which one has the most calories (kcal) per 100 grams (choose one)

- Product 1
- Product 2
- Both have the same quantity

Considering product 1, what are the sources of sugar in the ingredient list? (choose one)

- Sugar and malt syrup
- Sugar, fructose and lecithin
- Sugar, fructose and malt syrup

Which of these diseases is related to a low intake of fibre? (choose one)

- Bowel disorders
- Anaemia
- Tooth decay

Which of these diseases is related to how much sugar people eat? (choose one)

- High blood pressure
- Tooth decay
- Anaemia

Which of these diseases is related to how much salt (or sodium) people eat? (choose one)

- Hypothyroidism
- Diabetes
- High blood pressure

Which of these options do experts recommend to reduce the chances of getting cancer? (choose one)

- Drinking alcohol regularly
- Eating less red meat
- Avoiding additives in food

Which of these options do experts recommend to prevent heart disease? (choose one)

- Taking nutritional supplements
- Eating less oily fish
- Eating less trans-fats

Which of these options do experts recommend to prevent diabetes? (choose one)

- Eating less refined foods
- Drinking more fruit juice
- Eating more processed meat

Which one of these foods is more likely to raise people’s blood cholesterol? (choose one)

- Eggs
- Vegetable oils
- Animal fat

Which one of these foods is classified as having a high Glycaemic Index (Glycaemic Index is a measure of the impact of a food on blood sugar levels, thus a high Glycaemic Index means a greater rise in blood sugar after eating)? (choose one)

- Wholegrain cereals
- White bread
- Fruit and vegetables

To maintain a healthy weight people should cut fat out completely. (choose one)

- Agree
- Disagree

To maintain a healthy weight people should eat a high protein diet. (choose one)

- Agree
- Disagree

Eating bread always causes weight gain. (choose one)

- Agree
- Disagree

Fibre can decrease the chances of gaining weight. (choose one)

- Agree
- Disagree

Which of these options can help people to maintain a healthy weight? (answer each one with yes or no)

- Not eating while watching TV
- Reading food labels
- Taking nutritional supplements
- Monitoring their eating
- Monitoring their weight
- Grazing throughout the day

If someone has a Body Mass Index (BMI) of 23kg/m^2^, what would their weight status be? (choose one)

- Underweight
- Normal weight
- Overweight
- Obese

If someone has a Body Mass Index (BMI) of 31kg/m^2^, what would their weight status be? (choose one)

- Underweight
- Normal weight
- Overweight
- Obese

Which of these body shapes increases the risk of cardiovascular disease (Cardiovascular disease is a general term that describes a disease of the heart of blood vessels, for example, angina, heart attack, heart failure, congenital heart disease and stroke)? (choose one)

- Apple shape
- Pear shape
