# Supplementary material for: Comparative analysis of AI on human nutrition knowledge: Evaluating large language model-based conversational agents against dietetics students and the general population
Source: PLoS One. 2025 Dec 8;20(12):e0336577. doi: 10.1371/journal.pone.0336577 (PMC12685196; doi:10.1371/journal.pone.0336577)
Supplement: S2 File — (DOCX) [file pone.0336577.s002.docx]

**Supplementary Information 2. Replies provided by Large Language Models.**

| **ChatGPT-4** | **ChatGPT-3.5** | **Bard** | **Copilot** |
| --- | --- | --- | --- |
| **Section 1**   - **Fruit**: More - **Food and drinks with added sugar**: Less - **Vegetables**: More - **Fatty foods**: Less - **Processed red meat**: Less - **Wholegrains**: More - **Salty foods**: Less - **Water**: More - **5 or more servings** - **Unsaturated fats**: Not eat less - **Trans fats**: Eat less - **Saturated fats**: Eat less - **Reduced fat (e.g., skimmed and semiskimmed milk)** - **1-2 times per week** - **2 drinks for men and 1 drink for women** - **Every day** - **One serving** - **Third** | **Section 1**   - **Fruit**: More - **Food and drinks with added sugar**: Less - **Vegetables**: More - **Fatty foods**: Less - **Processed red meat**: Less - **Wholegrains**: More - **Salty foods**: Less - **Water**: More - **5 or more servings** - **Unsaturated fats**: Not eat less - **Trans fats**: Eat less - **Saturated fats**: Eat less - **Reduced fat (e.g., skimmed and semiskimmed milk)** - **1-2 times per week** - **2 drinks for men and 1 drink for women** - **Every day** - **One serving** - **Third** | **Section 1**   - **Fruit**: More - **Food and drinks with added sugar**: Less - **Vegetables**: More - **Fatty foods**: Less - **Processed red meat**: Less - **Wholegrains**: More - **Salty foods**: Less - **Water**: More - **5 or more servings** - **Unsaturated fats**: Not eat less - **Trans fats**: Eat less - **Saturated fats**: Not eat less - **Reduced fat (e.g., skimmed and semiskimmed milk)** - **1-2 times per week** - **2 drinks for men and 1 drink for women** - **Every day** - **One serving** - **Half** | **Section 1**   - **Fruit**: More - **Food and drinks with added sugar**: Less - **Vegetables**: More - **Fatty foods**: Less - **Processed red meat**: Less - **Wholegrains**: More - **Salty foods**: Less - **Water**: More - **5 or more servings** - **Unsaturated fats**: Not eat less - **Trans fats**: Eat less - **Saturated fats**: Eat less - **Reduced fat (e.g., skimmed and semiskimmed milk)** - **1-2 times per week** - **1 drink each for men and for women** - **Every day** - **One serving** - **Third** |
| **Section 2**   - **Diet cola drinks**: Low in added sugar - **Natural yoghurt**: Low in added sugar - **Ice cream**: High in added sugar - **Tomato ketchup**: High in added sugar - **Melon**: Low in added sugar - **Breakfast cereals**: High in salt - **Frozen vegetables**: High in salt - **Bread**: High in salt - **Baked beans**: High in salt - **Red meat**: Low in salt - **Canned soup**: Low in salt - **Oats**: High in fibre - **Bananas**: Low in fibre - **White rice**: Low in fibre - **Eggs**: Low in fibre - **Potatoes with skin**: High in fibre - **Pasta**: Low in fibre - **Poultry**: Good source of protein - **Cheese**: Good source of protein - **Fruit**: Not a good source of protein - **Baked beans**: Good source of protein - **Butter**: Not a good source of protein - **Nuts**: Good source of protein - **Cheese**: Not a starchy food - **Pasta**: Starchy food - **Potatoes**: Starchy food - **Nuts**: Not a starchy food - **Plantains**: Starchy food - **Olive oil**: Monounsaturated fat - **Butter**: Saturated fat - **Sunflower oil**: Polyunsaturated fat - **Eggs**: Cholesterol - **Biscuits, cakes and pastries** - **About the same** - **Fat** - **Higher in calories** | **Section 2**   - **Diet cola drinks**: Low in added sugar - **Natural yoghurt**: Low in added sugar - **Ice cream**: High in added sugar - **Tomato ketchup**: High in added sugar - **Melon**: Low in added sugar - **Breakfast cereals**: High in salt - **Frozen vegetables**: High in salt - **Bread**: High in salt - **Baked beans**: High in salt - **Red meat**: High in salt - **Canned soup**: Low in salt - **Oats**: High in fibre - **Bananas**: Low in fibre - **White rice**: Low in fibre - **Eggs**: Low in fibre - **Potatoes with skin**: High in fibre - **Pasta**: High in fibre - **Poultry**: Good source of protein - **Cheese**: Good source of protein - **Fruit**: Not a good source of protein - **Baked beans**: Good source of protein - **Butter**: Not a good source of protein - **Nuts**: Good source of protein - **Cheese**: Not a starchy food - **Pasta**: Starchy food - **Potatoes**: Starchy food - **Nuts**: Not a starchy food - **Plantains**: Starchy food - **Olive oil**: Monounsaturated fat - **Butter**: Saturated fat - **Sunflower oil**: Polyunsaturated fat - **Eggs**: Cholesterol - **Biscuits, cakes and pastries** - **About the same** - **Fat** - **Higher in calories** | **Section 2**   - **Diet cola drinks**: Low in added sugar - **Natural yoghurt**: Low in added sugar - **Ice cream**: High in added sugar - **Tomato ketchup**: High in added sugar - **Melon**: Low in added sugar - **Breakfast cereals**: Low in salt - **Frozen vegetables**: Low in salt - **Bread**: Low in salt - **Baked beans**: Low in salt - **Red meat**: High in salt - **Canned soup**: High in salt - **Oats**: High in fibre - **Bananas**: Low in fibre - **White rice**: Low in fibre - **Eggs**: Low in fibre - **Potatoes with skin**: Low in fibre - **Pasta**: High in fibre - **Poultry**: Good source of protein - **Cheese**: Good source of protein - **Fruit**: Not a good source of protein - **Baked beans**: Good source of protein - **Butter**: Not a good source of protein - **Nuts**: Good source of protein - **Cheese**: Not a starchy food - **Pasta**: Starchy food - **Potatoes**: Starchy food - **Nuts**: Not a starchy food - **Plantains**: Starchy food - **Olive oil**: Polyunsaturated fat - **Butter**: Monounsaturated fat - **Sunflower oil**: Polyunsaturated fat - **Eggs**: Polyunsaturated fat - **Biscuits, cakes and pastries** - **About the same** - **Fat** - **Higher in calories** | **Section 2**   - **Diet cola drinks**: Low in added sugar - **Natural yoghurt**: Low in added sugar - **Ice cream**: High in added sugar - **Tomato ketchup**: High in added sugar - **Melon**: Low in added sugar - **Breakfast cereals**: Low in salt - **Frozen vegetables**: Low in salt - **Bread**: Low in salt - **Baked beans**: Low in salt - **Red meat**: High in salt - **Canned soup**: High in salt - **Oats**: High in fibre - **Bananas**: Low in fibre - **White rice**: Low in fibre - **Eggs**: Low in fibre - **Potatoes with skin**: High in fibre - **Pasta**: Low in fibre - **Poultry**: Good source of protein - **Cheese**: Good source of protein - **Fruit**: Not a good source of protein - **Baked beans**: Good source of protein - **Butter**: Not a good source of protein - **Nuts**: Good source of protein - **Cheese**: Not a starchy food - **Pasta**: Starchy food - **Potatoes**: Starchy food - **Nuts**: Not a starchy food - **Plantains**: Starchy food - **Olive oil**: Monounsaturated fat - **Butter**: Saturated fat - **Sunflower oil**: Polyunsaturated fat - **Eggs**: Cholesterol - **Biscuits, cakes and pastries** - **About the same** - **Fat** - **Higher in calories** |
| **Section 3**   - **Natural yogurt** - **Carrot butternut and spice soup** - **Roast turkey, mashed potatoes, and vegetables** - **Tuna salad sandwich + fruit + low fat yogurt + water** - **Berry sorbet** - **Broccoli, carrot, and tomatoes** - **Thick cut chips** - **Herbs** - **Sautéing** - **Medium fat** - **Disagree** - **Product 1** - **Sugar, fructose, and malt syrup** | **Section 3**   - **Natural yogurt** - **Carrot butternut and spice soup** - **Roast turkey, mashed potatoes, and vegetables** - **Tuna salad sandwich + fruit + low fat yogurt + water** - **Berry sorbet** - **Broccoli, carrot, and tomatoes** - **Thick cut chips** - **Herbs** - **Sautéing** - **Medium fat** - **Disagree** - **Product 2** - **Sugar and malt syrup** | **Section 3**   - **Natural yogurt** - **Carrot butternut and spice soup** - **Roast turkey, mashed potatoes, and vegetables** - **Tuna salad sandwich + fruit + low fat yogurt + water** - **Berry sorbet** - **Broccoli, carrot, and tomatoes** - **Thick cut chips** - **Herbs** - **Sautéing** - **Medium fat** - **Disagree** - **Product 2** - **Sugar and malt syrup** | **Section 3**   - **Natural yogurt** - **Carrot butternut and spice soup** - **Roast turkey, mashed potatoes, and vegetables** - **Tuna salad sandwich + fruit + low fat yogurt + water** - **Berry sorbet** - **Broccoli, carrot, and tomatoes** - **Thin cut chips** - **Herbs** - **Sautéing** - **Medium fat** - **Disagree** - **Product 2** - **Sugar and malt syrup** |
| **Section 4**   - **Bowel disorders** - **Tooth decay** - **High blood pressure** - **Eating less red meat** - **Eating less trans-fats** - **Eating less refined foods** - **Animal fat** - **White bread** - **Disagree** - **Disagree** - **Disagree** - **Agree** - **Not eating while watching TV**: No - **Reading food labels**: Yes - **Taking nutritional supplements**: Yes - **Monitoring their eating**: Yes - **Monitoring their weight**: Yes - **Grazing throughout the day**: No - **BMI of 23kg/m^2^**: Normal weight - **BMI of 31kg/m^2^**: Obese - **Apple shape** | **Section 4**   - **Bowel disorders** - **Tooth decay** - **High blood pressure** - **Eating less red meat** - **Eating less trans-fats** - **Eating less refined foods** - **Animal fat** - **White bread** - **Disagree** - **Disagree** - **Disagree** - **Agree** - **Not eating while watching TV**: Yes - **Reading food labels**: Yes - **Taking nutritional supplements**: No - **Monitoring their eating**: Yes - **Monitoring their weight**: Yes - **Grazing throughout the day**: No - **BMI of 23kg/m^2^**: Normal weight - **BMI of 31kg/m^2^**: Obese - **Apple shape** | **Section 4**   - **Bowel disorders** - **High blood pressure** - **High blood pressure** - **Eating less red meat** - **Eating less trans-fats** - **Eating less refined foods** - **Animal fat** - **White bread** - **Disagree** - **Disagree** - **Disagree** - **Agree** - **Not eating while watching TV**: Yes - **Reading food labels**: Yes - **Taking nutritional supplements**: Yes - **Monitoring their eating**: Yes - **Monitoring their weight**: Yes - **Grazing throughout the day**: No - **BMI of 23kg/m^2^**: Normal weight - **BMI of 31kg/m^2^**: Overweight - **Apple shape** | **Section 4**   - **Bowel disorders** - **Tooth decay** - **High blood pressure** - **Eating less red meat** - **Eating less trans-fats** - **Eating less refined foods** - **Animal fat** - **White bread** - **Disagree** - **Disagree** - **Disagree** - **Agree** - **Not eating while watching TV**: Yes - **Reading food labels**: Yes - **Taking nutritional supplements**: Yes - **Monitoring their eating**: Yes - **Monitoring their weight**: Yes - **Grazing throughout the day**: No - **BMI of 23kg/m^2^**: Normal weight - **BMI of 31kg/m^2^**: Overweight - **Apple shape** |
